# More Robust Co-Occurrence Patterns and Stronger Dispersal Limitations of Bacterial Communities in Wet than Dry Seasons of Riparian Wetlands

**DOI:** 10.1128/msystems.01187-22

**Published:** 2023-03-23

**Authors:** Liyan Zhang, Yi Li, Xiangxin Sun, Jonathan M. Adams, Longfei Wang, Huanjun Zhang, Haiyan Chu

**Affiliations:** a Key Laboratory of Integrated Regulation and Resource Development on Shallow Lakes, Ministry of Education, College of Environment, Hohai University, Nanjing, China; b College of Resources and Environmental Sciences, Nanjing Agricultural University, Nanjing, China; c Department of Geography and Oceanography, Nanjing University, Nanjing, China; d State Key Laboratory of Soil and Sustainable Agriculture, Institute of Soil Science, Chinese Academy of Sciences, Nanjing, China; e University of Chinese Academy of Sciences, Beijing, China; Los Alamos National Laboratory

**Keywords:** ecological networks, community assembly, riparian wetland, potential functions, wet-dry periods, environmental vulnerability

## Abstract

Riparian wetlands can be used as “sentinels” of environmental changes and play pivotal roles in ecological and biogeochemical processes. The bacterial community is an essential and rapidly responding component in riparian areas. However, the co-occurrence patterns and phylogenetic group-based ecological processes during wet-dry periods are still open questions. Here, we compared the co-occurrence patterns and phylogenetic group-based assembly mechanisms of soil bacterial communities in typical riparian wetlands across wet and dry seasons, which are subjected to intensive agricultural activities. The results showed that the potential functions, community composition, network structure, and phylogenetic group-based ecological processes of the bacterial community were distinct between the wet and dry seasons. The stability and complexity of the wet season bacterial network were significantly higher than those of the dry season bacterial network. Moreover, the phylogenetic group-based null model analysis showed that homogeneous selection (HoS), dispersal limitation (DL), and drift (DR) were the most important ecological processes for the bacterial community assemblages, with a higher proportion of DL in the wet season (36.7%) than in the dry season (25.5%) but lower contributions of the HoS (36.1% versus 41.3%) and DR (20.8% versus 25.4%). The communities dominated by *Flavobacteriales*, *Burkholderiales*, and *Sphingomonadales* in the wet season were controlled more by dispersal limitation, whereas they were significantly negatively correlated with precipitation, dissolved organic carbon, and total carbon in the dry season, respectively. These findings expand our understanding of the network vulnerability and assembly mechanisms in fragile anthropologically affected riparian wetland ecosystems.

**IMPORTANCE** Riparian wetlands comprise microbial communities that are easily affected by the surrounding conditions, especially in agricultural landscapes with a wide range of ecosystem services. After comparing the wet and dry season microbiota, we found that the soil bacterial community of the wet season exhibited a higher complexity and stability of soil bacterial network and stronger dispersal limitations than that of the dry season; however, the dry season bacteria showed stronger homogeneous selection than the wet season bacteria. The co-occurrence and phylogenetic group-based bacterial community assembly mechanisms were mainly shaped by the divergence in temperature and precipitation between seasons. Revealing the variations in the potential functions, co-occurrence, and community assembly processes between wet and dry seasons is critical to understanding the maintenance of soil microbial diversity in riparian wetlands with regard to environmental sceneries.

## INTRODUCTION

Riparian wetlands are sentinels of environmental changes on Earth and play crucial roles in sustaining ecosystem functions and services between inland and the ocean (1, 2), which can mitigate diffuse pollution and provide inputs to adjacent ecosystems (3). Bacteria are versatile players in simultaneously driving multiple ecological functions, such as nutrient leaching, pollutant removal, and soil C and N cycling (4–7), and are an integral and quickly responding component in riparian zones (8). Increased natural and anthropogenic forces (e.g., climate change and agriculturalization) pose many impacts on lands, resulting in more complex wetland processes and highly variable biodiversity (9, 10). Riparian wetlands experience remarkable wet-dry cycles annually, leading to intermittent hydrological changes (11). During the wet period, such riparian soils are vulnerable to rain percolation, which affects biochemical processes, whereas in the dry season, water supply becomes a limiting factor for microbial attributes. Therefore, exploring the bacterial community during wet and dry hydrologic periods in riparian wetlands could advance forecasting of the responses of riparian wetland ecosystems to anthropogenic disturbance and thus help manage soil bacterial communities from scale-dependent benefits for better provisioning of ecosystem services and biodiversity.

The microbial ecological network (MEN) theory provides us with new insights into microbial food webs (12–14) and has been used in delineating the potential linkages between species in many ecosystems (15, 16). As precipitation levels, the water table, and nutrients (NO_3_^−^ concentrations) generally fluctuate throughout wet-dry periods (17, 18), the corresponding changes are reflected in the diversity and co-occurrence networks of bacterial communities (11). Microorganisms usually form ecological clusters (i.e., modules) within networks that are sensitive to abiotic and biotic factors (19, 20). However, we still lack a predictive understanding of the ecological attributes of bacterial communities in riparian wetlands during wet-dry periods and their environmental preferences. Understanding the correlation-based network patterns is essential for predicting bacterial ecological and evolutionary processes and the associated impacts on land loss, fragmentation, and soil degradation.

Understanding community assembly mechanisms is crucial for maintaining and promoting ecosystem functions and services (21, 22). It is generally accepted that deterministic and stochastic processes are both important in facilitating the assembly of microbial communities (23–25). Determinism is largely dictated by selection (e.g., competition and mutualism), environmental conditions, and interspecies interactions that govern community structure, whereas stochasticity is characterized by random ecological drift, dispersal, and speciation (24, 26). A conceptual framework including five ecological processes (homogeneous selection, heterogeneous selection, homogenizing dispersal, dispersal limitation, and drift) has been developed to disentangle their contributions. A previous study showed that ecological drift and dispersal limitation controlled communities dominated by *Proteobacteria* (e.g., *Sphingomonadales*) in regional riparian wetlands (27). In addition, it has been shown that homogenizing dispersal and dispersal limitation dominated soil bacterial communities in the fresh-saltwater transition zone of riparian wetlands (28). However, there was a slightly higher effect of stochasticity shaping the anthropogenically affected river communities in the wet season than in the dry season due to dynamic hydrologic alteration (11). In addition, it has been indicated that the assembly mechanisms at the group level were more accurate than at the “whole-community” level, as the group level has been shown to be superior in illustrating prokaryotic communities (29–31). However, there is still a gap in our ecological knowledge regarding phylogenetic group (PG)-based riparian soil bacterial assembly mechanisms.

**FIG 1:**
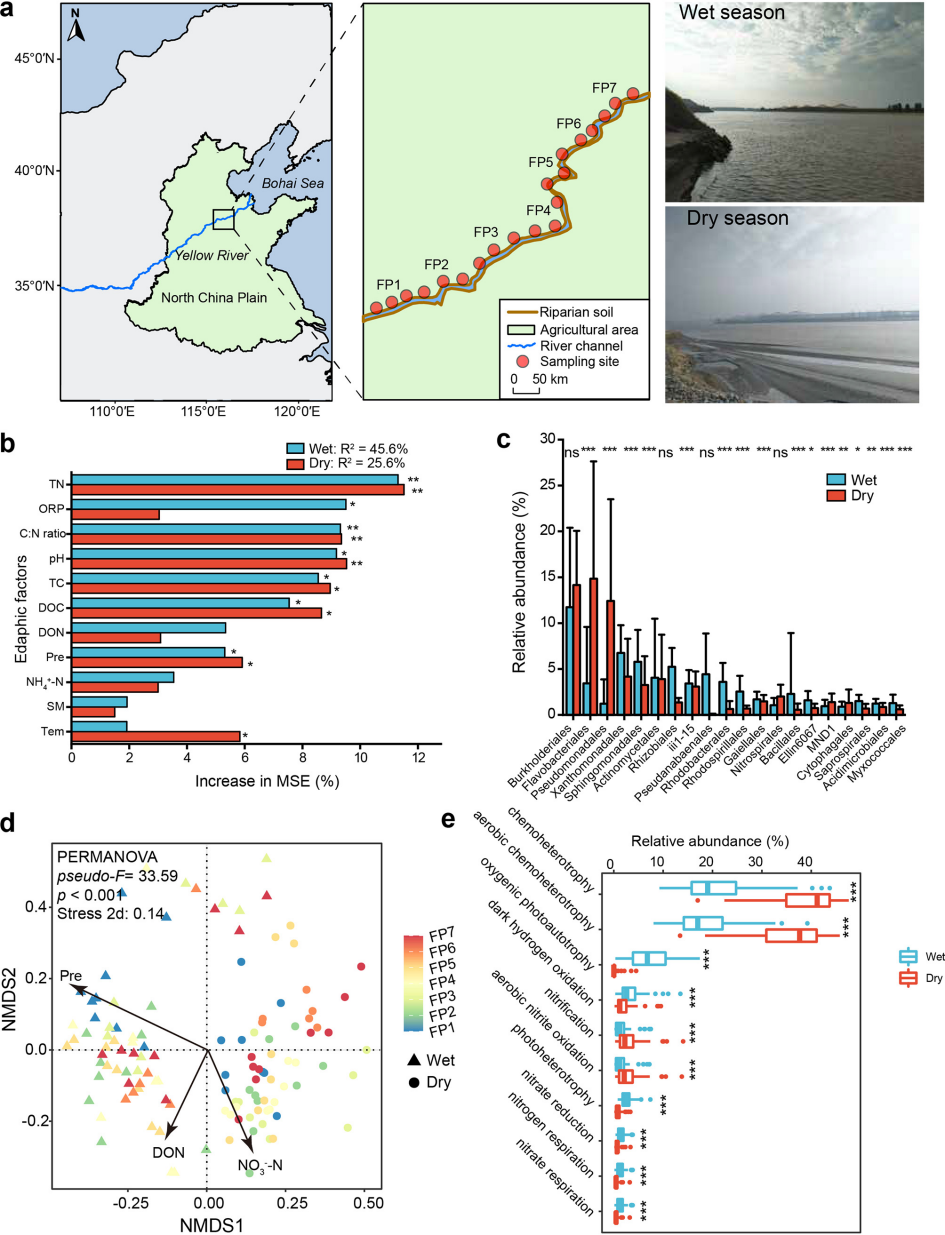
Summary of site locations and differences in the relative importance of soil edaphic factors on the diversity, abundance, and community structure of bacterial communities. (a) Sketch map showing the distribution of sampling sites; (b) relative importance of edaphic factors on observed operational taxonomy units (OTUs) between seasons; (c) comparison of the relative abundance of the top 20 bacterial orders between seasons. Significance was tested using linear regression analyses, and error bars show standard deviations of the mean. *, *P* < 0.05; **, *P* < 0.01; ***, *P* < 0.001; ns, *P* > 0.05. (d) Nonmetric multidimensional scaling (NMDS) plot of soil bacterial community dissimilarities based on the Bray-Curtis distance matrix. The three best environmental drivers were chosen by the *bioenv* function. (e) Relative abundance of 10 major potential functions in bacterial communities identified using the FAPROTAX database. Significant differences between seasons are marked: ***, *P* < 0.001. TN, total nitrogen; C:N ratio, carbon/nitrogen ratio; DOC, dissolved organic carbon; DON, dissolved organic nitrogen; Pre, precipitation; Tem, temperature; NH_4_^+^-N, ammonium nitrogen; SM, soil moisture.

To this end, we collected riparian soils along the North China Plain (NCP) of the lower Yellow River during the wet and dry seasons at 20 continuous sampling sites (Fig. 1a). The riparian wetlands in this region are subject to heavy agricultural stress (32). The NCP is situated on the second-most-populated plain on Earth and has a nearly three times higher population density (1.2 × 10^9^ in total with 1,000 to 10,000 people/km^2^) than the global average level (33). The NCP is also the major food production region in Asia, feeding 22% of the Chinese population (34); many of its riparian zones have been reclaimed to agricultural soil (35). The large seasonal variation in its water balance is a special feature of this zone. Precipitation is concentrated in the wet (i.e., flooding) season, which constitutes more than 60% of the annual discharge, while the dry season represents less than 20%. We aimed to test three hypotheses: (i) in the scenario of seasonal variability, soil bacterial community structure and potential functions would differ during wet and dry periods, (ii) the wet season bacterial co-occurrence patterns would be more complex than those in the dry season and the associated environmental vulnerabilities would differ between seasons, and (iii) soil bacterial communities are more controlled by homogeneous selection in the dry season than the wet season.

## RESULTS

### Driving forces in structuring bacterial diversity.

The observed soil edaphic properties are shown in Table S1 and Table S2 in the supplemental material. Eight out of 12 edaphic properties in the wet season were significantly different from those in the dry season (Tukey’s honestly significant difference [HSD] test; *P* < 0.05). Compared with the wet season, the temperature, precipitation, pH, ammonium nitrogen (NH_4_^+^-N), dissolved oxygen content (DOC), total carbon (TC), and carbon to nitrogen (C:N) ratio significantly decreased in the dry season (*P* < 0.05), while the nitrate nitrogen (NO_3_^−^-N) was significantly higher (Mann-Whitney test; *P* = 0.003) in the dry season. The soil moisture (SM), NO_3_^−^-N, and total nitrogen (TN) were not significantly different between the two seasons (Tukey’s HSD test; *P* < 0.05).

10.1128/msystems.01187-22.6 TABLE S1 Summary of environmental variables across wet and dry seasons. Download Table S1, XLSX file, 0.02 MB. Copyright © 2023 Zhang et al. 2023 Zhang et al. https://creativecommons.org/licenses/by/4.0/ This content is distributed under the terms of the Creative Commons Attribution 4.0 International license.

10.1128/msystems.01187-22.7 TABLE S2 Comparison of environmental factors between wet and dry seasons. Download Table S2, XLSX file, 0.01 MB. Copyright © 2023 Zhang et al. 2023 Zhang et al. https://creativecommons.org/licenses/by/4.0/ This content is distributed under the terms of the Creative Commons Attribution 4.0 International license.

The bacterial communities in the wet season showed higher species richness than those in the dry season. Moreover, higher α diversity was also observed in the wet season (Adonis test; *P* < 0.001) (see Fig. S2 in the supplemental material). The random forest (RF) analysis between soil edaphic factors and bacterial diversity showed that 10 environmental variables (TN, C:N ratio, pH, TC, DOC, DON, NH_4_^+^-N, SM, precipitation, and temperature) controlled the bacterial diversity, which explained 45.6% and 25.6% of the bacterial diversity in the wet and dry seasons, respectively (Fig. 1b; Fig. S3). In both seasons, TN was the best predictor, with percentage increases in the mean squared error (IncMSE) over 10% (operational taxonomic unit [OTU] richness, 11.76% versus 12.41%; phylogenetic diversity, 13.01% versus 11.56%).

10.1128/msystems.01187-22.2 FIG S2 Comparison of bacterial diversity (Shannon index, OTU richness, and phylogenetic diversity) across wet and dry seasons (a) and different floating pontoons (b). All values are presented as the mean ± SD. ***, *P* < 0.001; **, *P* < 0.01; *, *P* < 0.05; ns, not significant. Download FIG S2, TIF file, 0.9 MB. Copyright © 2023 Zhang et al. 2023 Zhang et al. https://creativecommons.org/licenses/by/4.0/ This content is distributed under the terms of the Creative Commons Attribution 4.0 International license.

10.1128/msystems.01187-22.3 FIG S3 Relative importance of edaphic factors on microbial phylogenetic diversity in wet and dry seasons. TN, total nitrogen; C:N ratio, carbon/nitrogen ratio; DOC, dissolved organic carbon; TC total carbon; Pre, precipitation; Tem, temperature; NH_4_^+^-N, ammonium nitrogen; DON, dissolved organic nitrogen. Download FIG S3, TIF file, 0.6 MB. Copyright © 2023 Zhang et al. 2023 Zhang et al. https://creativecommons.org/licenses/by/4.0/ This content is distributed under the terms of the Creative Commons Attribution 4.0 International license.

### Seasonal changes in bacterial community composition and potential functions.

The community composition and structure varied between the wet and dry seasons (Fig. 1c; Fig. S4). Specifically, 5 out of 20 top orders were significantly higher in the dry season than in the wet season, including *Flavobacteriales*, *Pseudomonadales*, *Nitrospirales*, MND1, and *Cytophagales*. The relative abundances of *Burkholderiales*, *Actinomycetales*, iii1-15, and *Gaiellales* exhibited little but no significant variances between seasons. Furthermore, both nonmetric multidimensional scaling (NMDS) and permutational multivariate analyses of variance (PERMANOVAs) showed that bacterial communities were significantly different between the two seasons (*P* < 0.001) (Fig. 1d), with precipitation, DON, and NO_3_^−^-N being the best predictors driving their community variation, explaining 55.07% of the observed variation.

10.1128/msystems.01187-22.4 FIG S4 Relative abundance of the top 20 orders in the wet and dry seasons. Download FIG S4, TIF file, 2.6 MB. Copyright © 2023 Zhang et al. 2023 Zhang et al. https://creativecommons.org/licenses/by/4.0/ This content is distributed under the terms of the Creative Commons Attribution 4.0 International license.

We further predicted the microbial ecological functions using the FAPROTAX database. Ten major functions were shared between the wet and dry season bacteria: chemoheterotrophy, aerobic chemoheterotrophy, oxygenic photoautotrophy, dark hydrogen oxidation, nitrification, aerobic nitrite oxidation, photoheterotrophy, nitrate reduction, and nitrogen and nitrate respiration. The relative abundances of aerobic chemoheterotrophy, chemoheterotrophy, nitrification, and aerobic nitrite oxidation were significantly (Mann-Whitney test; *P* < 0.001) higher in the dry season than in the wet season, while the relative abundance of oxygenic photoautotrophy, dark hydrogen oxidation, photoheterotrophy, nitrate reduction, and nitrogen and nitrate respiration was significantly (Mann-Whitney test; *P* < 0.001) higher in the wet season than in the dry season (Fig. 1e).

### Ecological network patterns vary between seasons.

**FIG 2:**
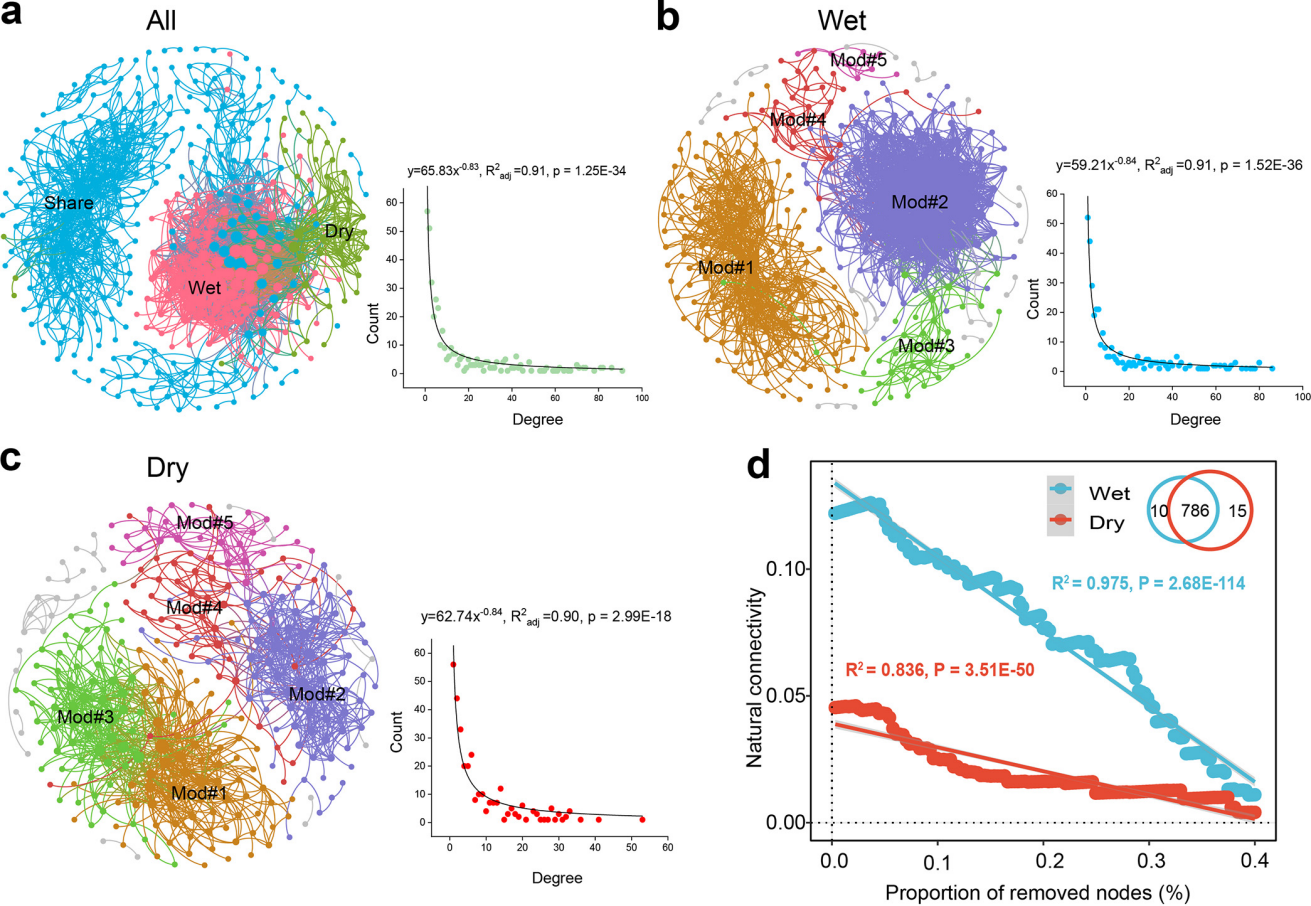
Ecological network visualization of the biotic associations in the bacterial community. Spearman’s correlation network between operational taxonomic units (OTUs) in all (a), wet (b), and dry (c) samples. A power law degree distribution pattern is illustrated in the MENs. A connection stands for a strong correlation coefficient of |*r*| > 0.80 and *P* < 0.01. The size of each node is proportional to the number of connections (i.e., degree). Only the top five modules (Mod 1 to 5) are shown in different colors, and other modules with <8 nodes per module are shown in gray. (d) Network structural robustness assessed by the decline in natural connectivity against the species of removed nodes.

The bacterial meta-network consisted of 400 nodes linked by 3,061 edges (Fig. 2a), which was mainly composed of taxa from the orders *Burkholderiales* (10.25%), *Sphingomonadales* (6.75%), *Xanthomonadales* (6.75%), *Pseudomonadales* (5.75%), *Pseudanabaenales* (4.75%), *Rhodobacterales* (4.5%), iii1-15 (4.25%), and *Rhizobiales* (4.0%). The meta-network was composed of 22%, 11%, and 67% wet season, dry season, and shared OTUs, respectively. Compared with the dry season bacterial network, the natural connectivity of the linear regression slope was always higher, indicating a more stable and robust bacterial network in the wet season (Fig. 2b and c). The empirical (real) network of bacterial communities in wet and dry seasons exhibited a better fit for power law distribution (*R*^2^ = 0.91 and 0.90 for wet and dry seasons, respectively) (Fig. 2) than the random networks (Erdös-Rényi model) (Table S3), suggesting that the self-organization of the ecological networks exhibited power law distributions for the degree (36). The topological properties in the empirical networks were higher than those in the respective Erdös-Réyni random networks, demonstrating that the networks had scale-free properties and modular structure.

10.1128/msystems.01187-22.8 TABLE S3 Topological properties of the bacterial co-occurrence networks and comparison between empirical and random networks in wet, dry, and both seasons. Download Table S3, XLSX file, 0.01 MB. Copyright © 2023 Zhang et al. 2023 Zhang et al. https://creativecommons.org/licenses/by/4.0/ This content is distributed under the terms of the Creative Commons Attribution 4.0 International license.

**FIG 3:**
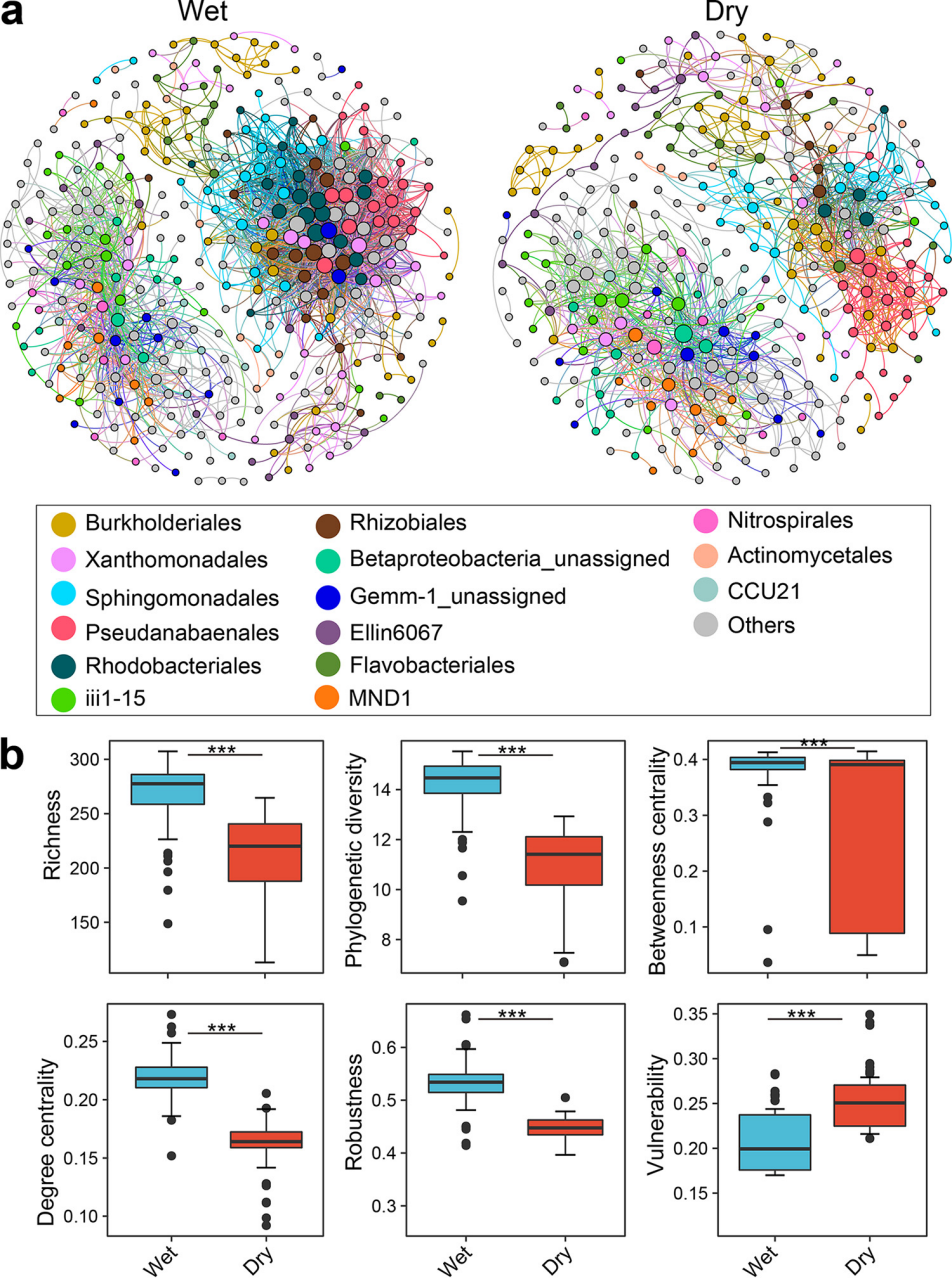
Taxonomic profiles and node-level topological features in the co-occurrence panel display. (a) The taxonomic profiles of bacterial communities at the order level in the co-occurrence panel display the wet and dry seasons (a). The nodes were colored according to the order level of the taxonomy. A connection stands for a strong correlation coefficient of |*r*| > 0.80 and *P* < 0.01. The size of each node is proportional to the number of connections (i.e., degree). (b) Comparison of node-level topological features between seasons. The top and bottom boundaries of each box indicate the 75th and 25th quartile values, respectively, and lines within each box represent the median values. ***, *P* < 0.001 (Wilcoxon rank sum test).

The ecological networks were clearly parsed into six major ecological clusters (modules [e.g.], Mod #1, Mod #2, etc.), which covered over 92% of the bacterial phylotypes. The wet season bacteria supported 356 nodes that were linked by 2,747 edges with phylogenetically diverse phylotypes in the wet season, whereas the dry season bacterial network was constructed by 312 nodes linked by 1,246 edges (Fig. 3a; Table S3). Accordingly, a higher proportion of positive links was observed between these modules in the wet season (94.3%) than in the dry season (93.42%), suggesting that the wet season had more closely associated bacterial co-occurrence patterns. We compared the node-level topological features (i.e., diversity, degree centrality, betweenness centrality, robustness, and vulnerability) between the two seasons (Fig. 3b). The richness, phylogenetic diversity, degree centrality, betweenness centrality, and robustness were all significantly higher (Wilcoxon rank sum test; *P* < 0.001) in the wet season than in the dry season. The network vulnerability, the maximum decrease in network efficiency when a node is extracted from the network, was also lower in the wet season than in the dry season, suggesting that the wet bacterial network was more stable and complex than the dry bacterial network.

**FIG 4:**
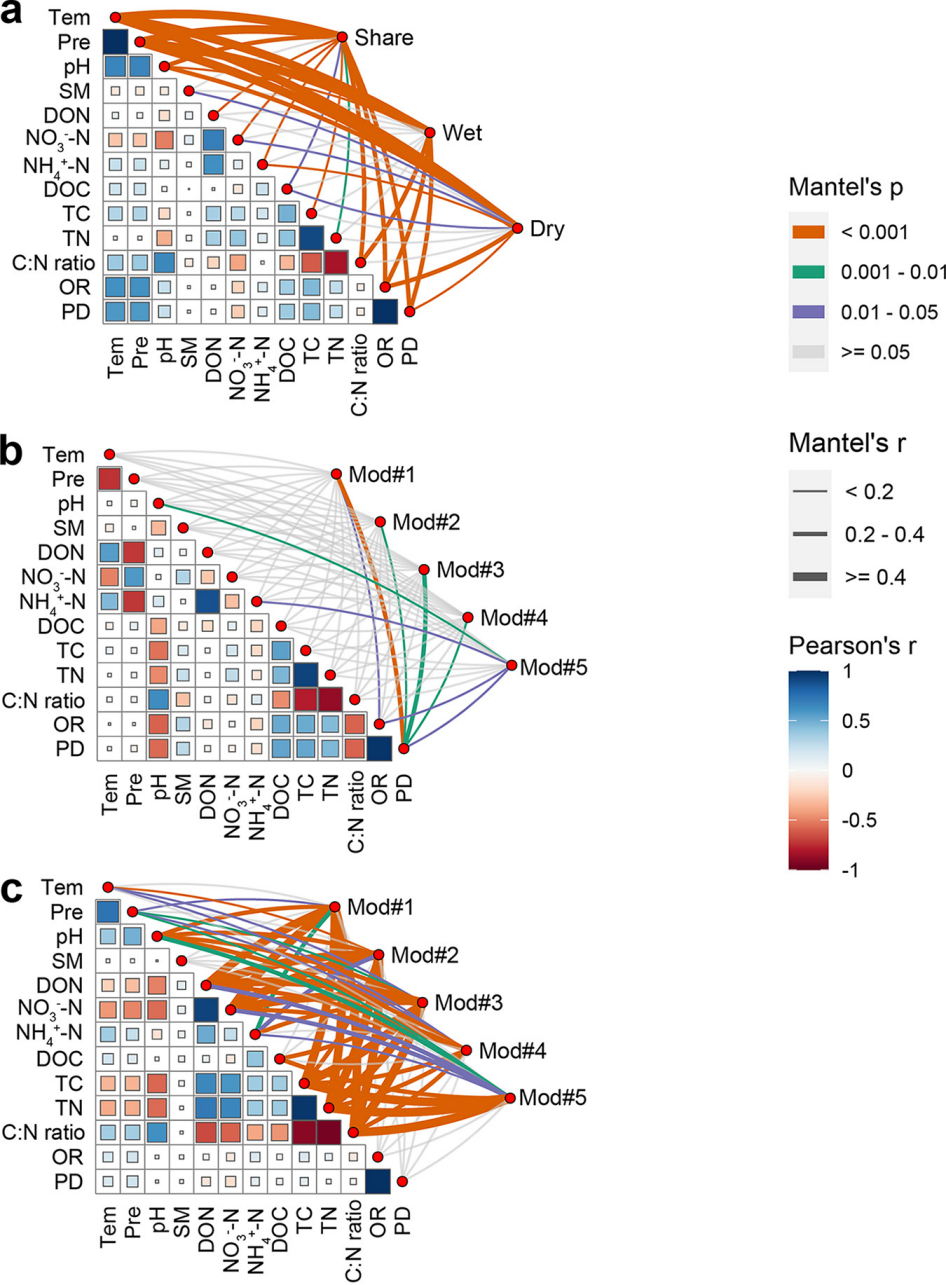
Pairwise comparisons between environmental variables, OTU richness, phylogenetic diversity and ecological modules (Mod #1 to Mod #5) in all (a), wet (b), and dry (c) seasons. The color gradient denotes Spearman’s correlation coefficients. Modules were related to each variable by the Mantel test. Edge width corresponds to Mantel’s *r* statistic for the corresponding distance correlations, and edge color denotes the statistical significance based on 9,999 permutations. Tem, temperature; Pre, precipitation; SM, soil moisture; DON, dissolved organic nitrogen; NO_3_^−^-N, nitrate nitrogen; NH_4_^+^-N, ammonium nitrogen; DOC, dissolved organic carbon; TC, total carbon; TN, total nitrogen; C:N ratio, carbon to nitrogen ratio; OR, OTU richness; PD, phylogenetic diversity.

We then linked edaphic factors, OTU richness, and phylogenetic diversity to the five ecological clusters in the individual networks (Fig. 4). For the whole bacterial community, temperature and precipitation were the most significant drivers of the bacterial networks. Moreover, the drivers controlling the major ecological clusters were distinct between the two seasons. For example, carbon and nitrogen content (e.g., TC, TN, C:N ratio, DON, and NO_3_^−^-N) exhibited strong correlates of community variation in the bacterial network of the dry season, such as Mod #1, Mod #3, and Mod #5, whereas bacterial α diversity (i.e., phylogenetic diversity) was most correlated with community variation in both Mod #1 and Mod #3 in the wet season.

### Variations in bacterial community assemblages.

**FIG 5:**
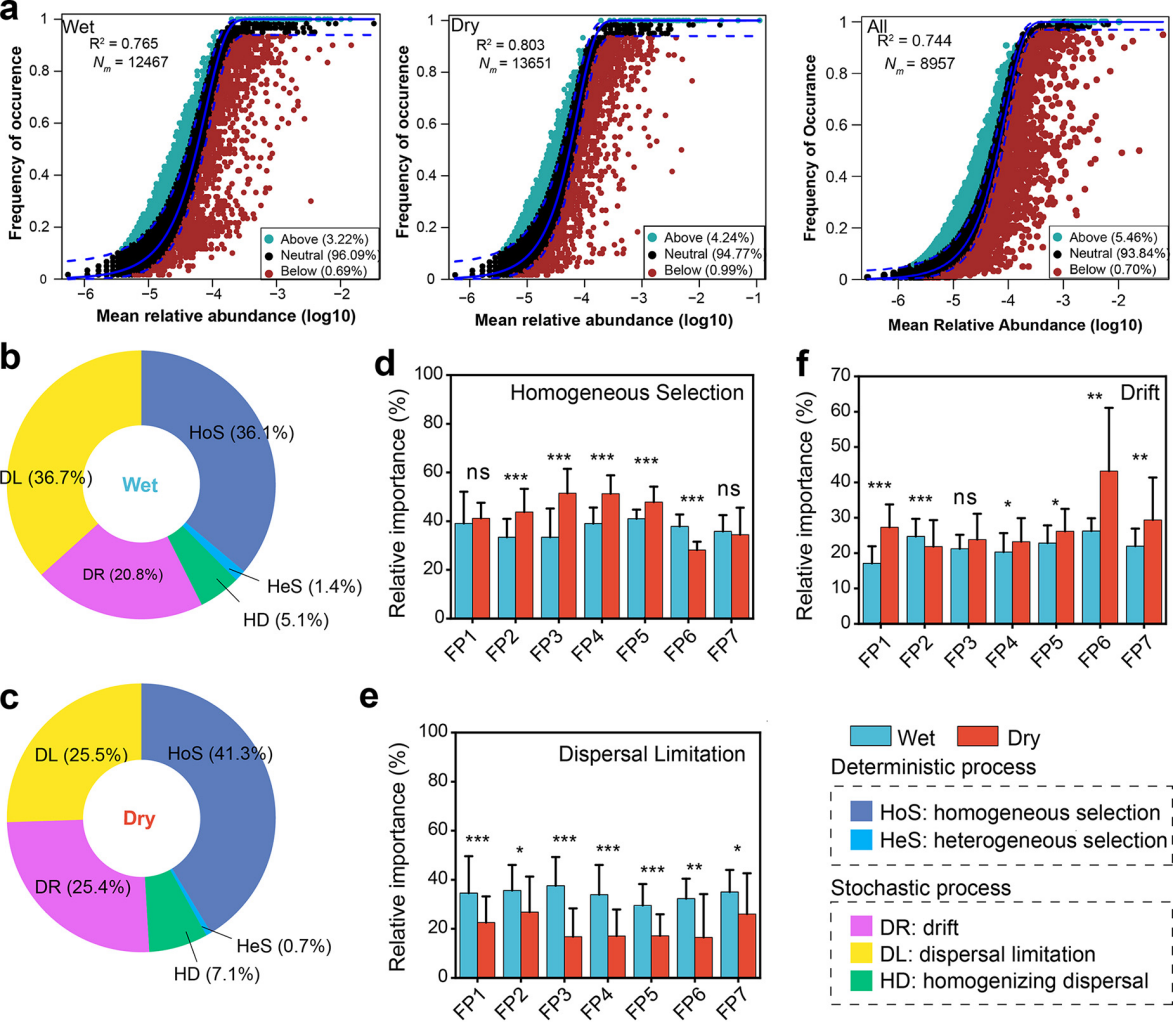
Relative importance of different ecological processes in governing bacterial communities. (a) Fit of the neutral community model (NCM) of community assembly. The solid blue lines indicate the best fit to the NCM, and the dashed blue lines represent 95% confidence intervals around the model prediction. OTUs that occur more or less frequently than predicted by the NCM are shown in different colors. *N_m_* indicates the metacommunity size × immigration, and *R*^2^ indicates the fit to this model. (b and c) Relative importance of different ecological processes during the wet (b) and dry (c) seasons; (d to f) comparison of changes in homogeneous selection (d), dispersal limitation (e), and drift (f) across the floating pontoons (FP1 to FP7) between wet and dry seasons. ***, *P* < 0.01; **, *P* < 0.05; *, *P* < 0.1; ns, not significant.

Neutral-theory-based modeling showed that 76.5%, 80.5%, and 74.4% of community variations were potentially explained by the neutral community during wet, dry, and both seasons, respectively (Fig. 5a). After quantifying the meta-community based on the phylogenetic group-based null model, we selected the top 7,000 OTUs in all samples, which accounted for 85% of the OTUs in all samples. The relative importance of the five fundamental ecological processes differed significantly across seasons and floating pontoons. Although both stochasticity and determinism controlled the bacterial community in the wet season, homogeneous selection (HoS) and dispersal limitation (DL) accounted for 36.1% and 36.7% of the community variation, respectively (Fig. 5b). However, HoS and DL accounted for 41.3% and 25.5% of the community variation during the dry season, respectively (Fig. 5c). In addition, we also detected a notable proportion of drift (DR) in the community variation, which accounted for 20.8% and 25.4% of the variation during the wet and dry seasons, respectively. The relative importance of DL was significantly higher in the wet season than in the dry season (Fig. 5d to f).

**FIG 6:**
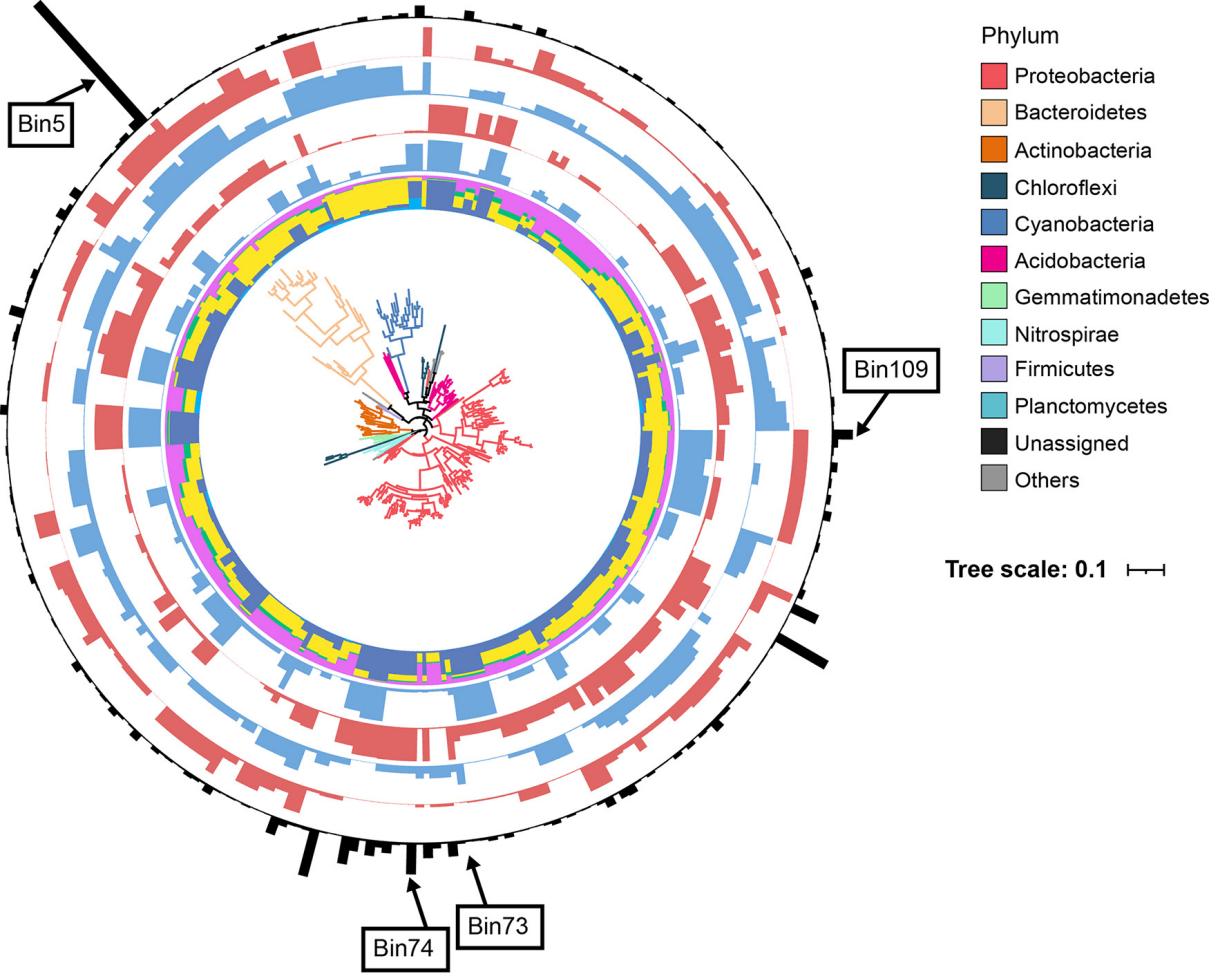
Variations in ecological processes across dominant bacterial taxa based on phylogenetic group-based null model analysis. The first annulus represents the relative importance of different ecological processes in each bin. Turquoise, dispersal limitation (DL); yellow, drift (DR); green, homogenizing dispersal (HD); sky blue, heterogeneous selection (HeS); rose red, homogeneous selection (HoS). The 2nd annulus and 3rd annulus represent the HoS in bin contribution during the wet and dry seasons, respectively. The 4th annulus and 5th annulus represent DL in bin contribution during the wet and dry seasons, respectively. The 6th annulus represents the relative abundance of each bin. Only the top 86 bins are shown in this figure, accounting for a total relative abundance of 60%. The size of each bar is proportional to the value of abundance (or contribution).

The top 7,000 OTUs were classified into 125 PGs on their phylogenetic relationships (Fig. 6; Fig. S5 and Table S4). The results showed that DL dominated 52 bins (41.6% of bin numbers and 39.2% of relative abundance), DR dominated 39 bins (31.2% of bin numbers and 20.9% of relative abundance), and HoS dominated 26 bins (20.8% of bin numbers and 27.6% of relative abundance) in the wet season. The major DL bin was *Flavobacteriales* (Bin5; 7.94% in total abundance of bins) in *Bacteroidetes*, the HoS bin was *Sphingomonadales* (Bin109; 3.39%) in *Alphaproteobacteria*, and the DR bin was *Burkholderiales* (Bin73; 2.00%) in *Betaproteobacteria*. In contrast, DR dominated 49 bins (39.2% of bin numbers and 29.8% of relative abundance), HoS dominated 42 bins (33.6% of bin numbers and 42.2% of relative abundance), and DL dominated 22 bins (17.6% of bin numbers and 20.2% of relative abundance) in the dry season (Table S5). The major DR bin was *Sphingomonadales* (Bin109; 3.37%) in *Proteobacteria*, the HoS bin was *Burkholderiales* (Bin74; 4.71%) in *Betaproteobacteria*, and the DL bin was *Flavobacteriales* (Bin5; 7.98%) in *Bacteroidetes*.

10.1128/msystems.01187-22.5 FIG S5 Variations in ecological processes across dominant bacterial taxa based on phylogenetic bin-based null model analysis. The first annulus represents the relative importance of different ecological processes in each group. Turquoise, dispersal limitation (DL); yellow, drift (DR); green, homogenizing dispersal (HD); sky blue, heterogeneous selection (HeS); rose red, homogeneous selection (HoS). The 2nd, 3rd, 4th, and 5th annuli represent the HoS and DL in bin contributions in the wet and dry seasons, respectively. The 6th annulus represents the relative abundance of each bin. The size of each bar is proportional to the value of abundance. Download FIG S5, TIF file, 1.2 MB. Copyright © 2023 Zhang et al. 2023 Zhang et al. https://creativecommons.org/licenses/by/4.0/ This content is distributed under the terms of the Creative Commons Attribution 4.0 International license.

10.1128/msystems.01187-22.9 TABLE S4 Relative abundance of each bin during the wet, dry, and both seasons. Download Table S4, XLSX file, 0.06 MB. Copyright © 2023 Zhang et al. 2023 Zhang et al. https://creativecommons.org/licenses/by/4.0/ This content is distributed under the terms of the Creative Commons Attribution 4.0 International license.

10.1128/msystems.01187-22.10 TABLE S5 Spearman's rho correlation between environmental variables against the relative importance of each ecological process in shaping bacterial communities. Download Table S5, XLSX file, 0.01 MB. Copyright © 2023 Zhang et al. 2023 Zhang et al. https://creativecommons.org/licenses/by/4.0/ This content is distributed under the terms of the Creative Commons Attribution 4.0 International license.

We further determined the contribution of the environmental variables mediating the bacterial assembly processes (Table 1), which showed that precipitation and temperature were the two most significant determinants of HoS and DL for the whole bacterial community. The relative abundances of the three major groups *Flavobacteriales*, *Burkholderiales*, and *Sphingomonadales* were not significantly correlated with any observed environmental factors during the wet season, whereas during the dry season, the relative abundance of *Flavobacteriales* was significantly negatively correlated with precipitation, and the relative abundances of *Burkholderiales* and *Sphingomonadales* were significantly negatively correlated with DOC and TC, respectively (Table 2).

**TABLE 1:** Spearman's rho correlation between environmental variables against the relative importance of each ecological process in shaping bacterial communities^*a*^

Variable	Homogeneous selection			Dispersal limitation			Drift		
	Wet	Dry	Total	Wet	Dry	Total	Wet	Dry	Total
Temperature	−0.013	0.052*	−0.541***	0.057*	0.052*	0.444***	−0.027	−0.067**	0.013
Precipitation	0.016	0.126***	−0.520***	0.068	0.036	0.484***	−0.038	−0.100***	−0.071***
pH	0.014	0.194***	−0.234***	0.039	0.165***	0.138***	−0.003	−0.219***	0.024*
SM	−0.011	0.053*	−0.06***	−0.058*	0.084***	0.061***	0.093***	−0.114***	−0.036**
DOC	0.007	−0.007	−0.029*	−0.042	0.126***	0.040***	0.100***	−0.198***	0.029*
DON	0.042	0.201***	−0.141***	−0.012	0.154***	0.079***	−0.006	−0.289***	0.02
NH_4_^+^-N	0.037	0.136***	−0.152***	0.0006	0.131***	0.083***	−0.029	−0.285***	0.053***
NO_3_^−^-N	−0.038	0.182***	−0.126***	0.01	0.131***	0.035**	0.083***	−0.298***	0.038**
TC	0.015	0.198***	−0.090***	0.084***	0.143***	0.124***	−0.005	−0.317***	−0.066***
TN	−0.017	0.247***	−0.095***	0.017	0.208***	0.095***	0.085***	−0.406***	−0.039***
C:N ratio	0.015	0.285***	−0.210***	0.046	0.266***	0.160***	0.026	−0.428***	−0.018

a SM, soil moisture; DOC, dissolved organic carbon; DON, dissolved organic nitrogen; NH_4_^+^-N, ammonium nitrogen; NO_3_^−^-N, nitrate nitrogen; TC, total carbon; TN, total nitrogen; C:N ratio, carbon to nitrogen ratio. The significance levels are noted as follows: *, *P* < 0.05; **, *P* < 0.01; and ***, *P* < 0.001.

**TABLE 2:** Influence of environmental variables on the abundances of dominant bacterial taxa by multiple regression on the distance matrix^*a*^

Variable	*Flavobacteriales*		*Burkholderiales*		*Sphingomonadales*	
	Wet (0.029)	Dry (0.032)	Wet (0.032)	Dry (0.222***)	Wet (0.034)	Dry (0.087*)
Temperature	0.098	0.064	−0.011	0.058**	−0.036	−0.037
Precipitation	−0.155	−0.112**	−0.063	−0.073***	0.012	−0.022
pH	−0.069	−0.04	−0.076	0.011	0.023	0.012
SM	0.02	−0.034	0.069	0.017	−0.037	0.017
DOC	−0.073	0.073	0.02	−0.144***	0.02	−0.102
DON	−0.155	0.037	−0.016	−0.002	0.009	0.002
NH_4_^+^-N	0.023	−0.003	−0.016	−0.002	−0.008	0.045
NO_3_^−^-N	0.149	0.006	0.001	0.002	0.022	0.084
TC	−0.058	−0.007	0.021	−0.081**	−0.032	−0.242**
TN	0.39	−0.146	0.261	0.072	0.121	0.313
C:N ratio	0.096	−0.006	0.022	0.068**	−0.028	0.158**

a SM, soil moisture; DOC, dissolved organic carbon; DON, dissolved organic nitrogen; NH_4_^+^-N, ammonium nitrogen; NO_3_^−^-N, nitrate nitrogen; TC, total carbon; TN, total nitrogen; C:N ratio, carbon to nitrogen ratio. The significance levels are noted as follows: *, *P* < 0.05; **, *P* < 0.01; ***, *P* < 0.01.

## DISCUSSION

### Dynamics of the bacterial community.

Riparian wetlands are naturally dynamic and diverse ecosystems. However, there are limited studies regarding their microbial ecology, co-occurrence patterns, and community assembly processes since these areas are always overlooked, especially in typical regions that often suffer from agricultural practices. The riparian ecosystems on the NCP have been influenced by intensive agricultural practices (35), which strongly impacted bacterial community dynamics during the wet and dry seasons in our study (Fig. 1). As expected, we found that riparian soil bacterial diversity was higher in the wet season than in the dry season, with divergent community composition and structure annually during the wet-dry cycles (PERMANOVA; *P* < 0.001). This can be explained by the variance in certain environmental variables, such as the temperature, precipitation, and carbon and nitrogen contents (see Table S2 in the supplemental material), and similar patterns were observed in the archaeal community structure of coastal surface sediment in eastern Chinese marginal seas (37). On the other hand, intermittent aridification could disturb nutrient sequestration, turnover, and transport (38). The dry season bacteria harbored a higher relative abundance of aerobic chemoheterotrophy, chemoheterotrophy, nitrification, and aerobic nitrite oxidation than the wet season, suggesting the roles of microbiota in improving nutrient resource acquisition (39). However, the relative abundances of oxygenic photoautotrophy, dark hydrogen oxidation, photoheterotrophy, nitrate reduction, and nitrogen and nitrate respiration were high in the wet season, which may suggest a sufficient nutrient source. Our results present evidence that soil bacterial community composition, potential functions, environmental vulnerabilities, and assembly mechanisms could change during wet-dry cycles in intensively agriculturally affected riparian wetlands.

### Dynamics of bacterial co-occurrence patterns and associated environmental preferences.

Exploring MENs across wet and dry seasons could expand our knowledge in understanding potential biotic interactions, habitat affinities, or shared environmental vulnerability under global climate change (14, 20). Overall, anthropogenic activity is associated with shifts in bacterial community composition, diversity, and soil food webs (8). In this study, we found that the empirical co-occurrence networks in both seasons were significantly distinct from the corresponding random networks, and a higher complexity and stability of the soil bacterial network were identified in the wet season. These characteristics may reflect past ecological and evolutionary dynamics and suggest a balance between niche and neutral models of community assembly processes (40–42).

The intensity of agricultural practices in our sampling areas obviously differed between seasons. In the wet season, frequent precipitation, high temperature, and agricultural practices increased surface runoff (43), which together changed the microbial food webs and enhanced soil bacterial interactions, whereas the bacterial alliance during the dry season formed relatively simple co-occurrence patterns to resist environmental stress (Fig. 3 and 4; Table S3), which may be due to the divergence of bacterial niche breadths and heterogeneity across wet-dry cycles annually, underpinned by consistent observations for the bacterioplankton communities in Taihu Basin (11). The distinct relationships between environmental modulators and ecological clusters may reflect their vulnerability in highly organized modules (20), which has been verified by linking environmental modulators, diversity and ecological modules (Fig. 4). Here, we found that the carbon and nitrogen sources (e.g., TC, TN, C:N ratio, DON, and NO_3_^−^-N) exhibited strong correlates of ecological clusters in the dry season but were not observed in the wet season. This result was in agreement with the fact that C and N are well recognized as critical factors for bacterial taxa due to their impact on bacterial activity and the specific selection of distinct bacterial lineages (44, 45).

### Contrasting bacterial community ecological processes during wet-dry periods.

Most prior studies have focused on the assembly processes of bacterial communities in lentic or lotic water ecosystems (46, 47), and far less is known about the assembly processes of bacterial communities in more complex and vulnerable anthropologically affected riparian wetland ecosystems. In this study, the bacterial community exhibited distinct seasonal dynamics, indicating that the assembly process might vary during wet-dry periods. The neutral community model and phylogenetic group-based null model (29) provide comprehensive and integrative insights into the dynamics of the bacterial community assembly mechanisms during wet-dry periods. These results demonstrated that both determinism and stochasticity shaped soil bacterial communities in riparian wetlands (Fig. 5, 6) but with a divergent proportion between the wet and dry seasons.

In this study, after quantifying community assembly into five ecological processes, we found that the contribution of HoS was lower in the wet season (36.1%) than in the dry season (41.6%), which demonstrated that determinism outweighed stochasticity in the dry season. The dry season is characterized by low water levels, slower and fewer hydrological regimens, and less fluidity (48), which drives similar community composition and hence increases the process of homogeneous selection. Similar results were found in freshwaters (49, 50). In addition, homogeneous selection (deterministic processes) was more pronounced during the dry season, a period during which droughts limit overall habitat availability and may thus act as a natural environmental filter (51).

The higher precipitation amounts and temperatures may act as environmental constraints which promoted greater dispersal limitation, possibly due to the wetter substrates introducing greater amounts of environmental heterogeneity (Fig. 1d); thus, communities are more likely to result in greater dispersal limitation (52). In our study, the relative abundance of the *Burkholderiales* in *Betaproteobacteria* governed by HoS increased with decreasing DOC (Table 2), and the relative abundance of *Sphingomonadales* in *Alphaproteobacteria* governed by DR increased with decreasing TC, indicating that *Burkholderiales* and *Sphingomonadales* may play vital roles in maintaining the diversity of soil bacterial communities during the dry season. Recently, studies have shown that some species (e.g., *Sphingomonas* spp. and *Burkholderiales* spp.) have roles in organometallic compound degradation, plant growth promotion, and stress tolerance (i.e., drought) in agriculturally affected soils (53, 54). These microorganisms are known to thrive in oligotrophic environments, such as carbon-limited marine environments (55), which may explain the substantial increase in their relative abundances with limited carbon sources. However, seasonal wet-dry cycling is an annually repeated process, and whether these ecologically significant taxa fit such alternative hydrologic processes requires long-term investigation.

### Implications for riparian wetland ecosystem management.

Anthropogenic disturbances cause habitat loss, and thus biodiversity is declining at an unprecedented rate. As the key ecosystems for regulating aquatic-terrestrial linkages, riparian wetlands are more vulnerable to anthropogenic disturbances (56, 57). The seasonal dynamics of soil bacterial co-occurrence patterns and community assembly processes can reflect ecosystem stability in the face of alternating wet-dry cycles. Species co-occurrence interactions favoring unproductive species increased biodiversity across temporal scales by decreasing selection effects (58), suggesting that species interactions can promote biodiversity and ecosystem services. Our findings indicated that periodic wet-dry cycles can be a stress that exerts a selective pressure disturbing bacterial diversity and altering bacterial community assembly mechanisms. This effect may be due to the divergence of precipitation amounts and temperatures during the wet season. Traditional management practices focus on improving local abiotic variables to increase local biodiversity but ignore dispersal across sites and biotic interactions, a concept that should be revisited.

### Conclusions.

This study advances the field by demonstrating that the soil bacterial community co-occurrence patterns, environmental vulnerabilities, and phylogenetic group-based community assembly mechanisms changed across wet and dry seasons in riparian wetlands. A more robust and stable co-occurrence network was observed in the wet season than in the dry season, suggesting that the wet season bacterial network was more complex. Moreover, the phylogenetic group-based null model indicated a season-dependent balance between stochasticity and determinism governing soil bacterial communities between wet and dry periods. Compared with that of the dry season bacterial community, the dispersal limitation was higher in wet season but made lower contributions to homogeneous selection and drift. The wet season bacterial communities dominated by *Flavobacteriales*, *Burkholderiales*, and *Sphingomonadales* were more controlled by dispersal limitation, whereas they were significantly negatively correlated with precipitation, dissolved organic carbon, and total carbon in the dry season, respectively. Taken together, our results provide clear evidence that the soil bacterial assemblages exhibited contrasting patterns between wet and dry seasons in riparian wetlands. These results highlight the need to take measures to manage riparian wetlands, which could improve our understanding of the microbial community assembly mechanisms underlying vulnerability to anthropogenic disturbances and ecosystem service maintenance under climate scenarios.

## MATERIALS AND METHODS

### Study area description and soil sampling.

The Yellow River originates on the Qinghai-Tibet Plateau (QTP) and flows to the northeast across the NCP before flowing into the Bohai Sea. We chose 20 continuous sampling locations across seven floating pontoons (FP1 to FP7) along the riparian wetlands (116°21′11″ to 116°41′22″ E, 36°18′24″ to 36°36′28″ N) (Fig. 1a; see Table S1 in the supplemental material). This area has a temperate zone monsoon climate, with a mean annual temperature of 11.7 to 12.6°C and a mean annual precipitation of 530 to 630 mm. At each sampling location, we selected three sampling plots with an interval of 2.5 km^2^ representing three replications. At each sampling plot, a 50- by 50-m sampling site was selected, and multipoint surface soil samples (0 to 25 cm) were randomly collected and blended into one sample. Finally, a total of 120 riparian soil samples were collected in August 2021 and January 2022, representing the wet (*n* = 60) and dry (*n* = 60) seasons, respectively. Sampling coordinate locations were recorded using a portable global positioning system (GPS Jisibao G138BD, Beijing, China). All samples were transported to the laboratory in an ice cooler and divided into two parts: one part was used for physiochemical analysis, and the other part was held at −20°C prior to DNA extraction.

### Physiochemical characteristics.

Soil pH was measured from soil-water suspensions (1:5 [wt/vol]). Soil moisture (SM) was determined gravimetrically by drying 10 g of the soil in an oven at 105°C for 24 h. Soil total carbon (TC) and total nitrogen (TN) were determined by the high-temperature combustion method and measured using an elemental analyzer (Vario MAX; Elementar, Germany). The dissolved total nitrogen (DTN), ammonium nitrogen (NH_4_^+^-N), and nitrate nitrogen (NO_3_^−^-N) were extracted from a mixture with a soil-potassium chloride solution (2 M) ratio of 1:10 suspensions and prefiltered through a 1.2-μm-pore sterile membrane. The leaching content was analyzed with a continuous flow analyzer (San++ system; Skalar, Holland). The dissolved organic nitrogen (DON) was calculated using the formula DON = DTN – (NH_4_^+^-N + NO_3_^−^-N) (Table S1).

### Illumina sequencing and molecular analyses.

Genomic DNA was extracted using a FastDNA spin kit (MoBio Laboratories, Inc., Carlsbad, CA, USA) according to the manufacturer’s guidelines. DNA concentration and purity were determined using a NanoDrop 2000 UV-visible (UV-Vis) spectrophotometer (Thermo Fisher, Wilmington, MA, USA). The V4-V5 region of the bacterial 16S rRNA (rRNA) gene was amplified using the primer pair 515F/907R. The oligonucleotide barcodes were fused to the 5′ end of the forward primer. PCR amplifications were performed on an ABI7500 Applied Biosystems (Thermo Fisher, USA) and prepared in triplicate. The PCR program conditions were as follows: an initial denaturation at 95°C for 3 min, followed by 29 cycles of denaturation at 95°C for 30 s, annealing at 53°C for 30 s, and extension at 72°C for 45 s, and then ending with a final extension at 72°C for 10 min. One microliter of 10 ng of template DNA from each soil sample was amplified in a 50-μL PCR mixture containing 10 μL (1.25 μM) of deoxynucleoside triphosphate (dNTP), 25 μL of Premix *Taq* (TaKaRa; catalog no. RR901), and 0.5 μL each of forward and reverse primers. Reaction yields were purified using DNA Clean-Up kits (Mo Bio, Carlsbad, CA, USA), pooled in equal molar amounts, and sequenced on an Illumina platform (Illumina, San Diego, CA, USA).

### Bioinformatics.

Raw sequences were processed and analyzed using the Quantitative Insights Into Microbial Ecology (QIIME) pipeline (59) for initial quality control, barcode extraction, and sequence merging. Amplicons with chimeric sequences were removed using VSEARCH 2.7.1 (60). Finally, we obtained a total of 5,515,543 sequences ranging from 30,082 to 84,702 counts per sample (with a median depth of 43,655). The sequences were clustered into operational taxonomic units (OTUs) at a 97% identify threshold using UPARSE v.7.1 (61). The obtained representative sequences were searched against the SILVA 132 database for taxonomic assignment (62) and aligned with the command *align_seqs.py* using PyNAST (63). A phylogenetic tree was constructed using FastTree (64). Singletons, doubletons, and OTUs that were present in only one sample were discarded as they were the potential artifact (65). Chloroplasts, archaea, and OTUs with fewer than 5 sequences were removed. To this end, we obtained a high-quality sequence data set yielding 44,296 OTUs comprising 3,232,267 clean reads across all samples. To fairly compare all of the samples at the same sequencing depth, the sequences were rarefied to 30,082 with a range of 2,450 to 7,256 OTUs per sample, with the rarefaction curves for individual and combined sets of all samples tending to approach saturation (see Fig. S1 in the supplemental material). Potential ecological functions of bacterial communities were identified using the FAPROTAX 1.2.6 database (66).

10.1128/msystems.01187-22.1 FIG S1 Rarefaction curves of operational taxonomic units (OTUs) at the 97% sequence similarity level for the individual samples (a) and the combined set of all samples (b). Download FIG S1, TIF file, 0.8 MB. Copyright © 2023 Zhang et al. 2023 Zhang et al. https://creativecommons.org/licenses/by/4.0/ This content is distributed under the terms of the Creative Commons Attribution 4.0 International license.

### Statistical analysis.

Comparisons of soil physiochemical properties between compartments were performed using the nonparametric Mann-Whitney test in SPSS Statistics 23.0 (IBM Corporation, Armonk, NY, USA). Comparisons of α diversity (Shannon index, OTU richness, and phylogenetic diversity) between different seasons were performed using the Adonis test in the vegan package. The relative importance of environmental variables on soil bacterial α diversity was calculated using the random forest (RF) machine learning method in the rfPermute package (67). The β diversity (based on the Bray-Curtis matrix) for each pairwise sample was calculated in the vegan package and visualized using nonmetric multidimensional scaling (NMDS) ordination. To screen the best set of abiotic environmental predictors, the *bioenv* function in the vegan package was used and fitted to the NMDS ordination.

Microbial ecological networks were constructed based on Spearman’s pairwise correlations of OTUs. To reduce the complexity, OTUs present in at least 20% of all samples (with a total of 811 OTUs) were kept. Spearman correlations between all OTUs were calculated by the WGCNA package, and OTU pairs with a significant correlation (|ρ| > 0.8; *P* < 0.01) were extracted and visualized with the interactive Gephi v.0.9.2 platform (https://gephi.org/) (68). The *P* values were adjusted using Benjamini-Hochberg’s false-discovery rate (FDR) controlling procedure to reduce the chance of false-positive results (69). The empirical and random networks of node-level topological properties were evaluated in the igraph package (70) and compared using the nonparametric Wilcoxon rank sum test in IBM SPSS Statistics 23.0 (SPSS, Inc., Cary, NC, USA). Pairwise comparisons between environmental factors, α diversity, and ecological clusters were assessed using the Mantel test.

We employed null-theory-based modeling (71) by predicting the relationship between occupancy and abundance (72). The fit of *R*^2^ was calculated using the minpack.lm package (73), and 95% confidence intervals (CIs) around the binomial proportion model predictions were calculated using the Wilson score interval in the HMisc package (74). We further used a phylogenetic bin-based (i.e., phylogenetic group [PG]) null model in the IEG Statistical Analysis Pipeline (http://ieg3.rccc.ou.edu:8080/root/login?redirect=%2F) to determine the significance of ecological assembly processes in governing the community based on the within-bin β-net relatedness index (βNRI) (29). Specifically, if the observed community turnover value |βNRI| was >1.96, the community was governed by deterministic processes (where βNRI < −1.96 represents homogenous selection and βNRI > 1.96 represents heterogeneous selection) (75, 76). If taxonomic diversity was different, as expected by random chance (|βNRI| ≤ 1.96), stochastic processes dictated community assembly. To further quantify the stochastic discrepancies, Raup-Crick dissimilarity (based on the Bray-Curtis matrix [RC_Bray_]) was employed to estimate the number of co-occurring species with species occurrence probabilities proportional to species frequencies between pairwise comparisons. Pairwise comparisons between communities that did not deviate from the null model were evaluated as the contribution of dispersal limitation (|βNTI| < 2 and RC_Bray_ > +0.95) and homogenizing dispersal (|βNTI| < 2 and RC_Bray_ < −0.95); RC_Bray_ values between −0.95 and +0.95 indicate that compositional turnover between pairwise communities is drift (77). The top 125 bins (accounting for a total relative abundance of 80.65%) were selected to construct a phylogenetic tree using FastTree and visualized on the iTOL (Interactive Tree Of Life) platform (https://itol.embl.de/) (78). Multiple-regression analysis was used to determine the relative importance of environmental variables in structuring the taxa in top bins using the ecodist package (79).

### Data availability.

The sequences have been deposited in the National Center for Biotechnology Information (NCBI) Sequence Read Archive (SRA) under BioProject no. PRJNA861733.
