# Supplementary figures and images for: More Robust Co-Occurrence Patterns and Stronger Dispersal Limitations of Bacterial Communities in Wet than Dry Seasons of Riparian Wetlands
Source: mSystems. 2023 Mar 23;8(2):e01187-22. doi: 10.1128/msystems.01187-22 (PMC10134804; doi:10.1128/msystems.01187-22)

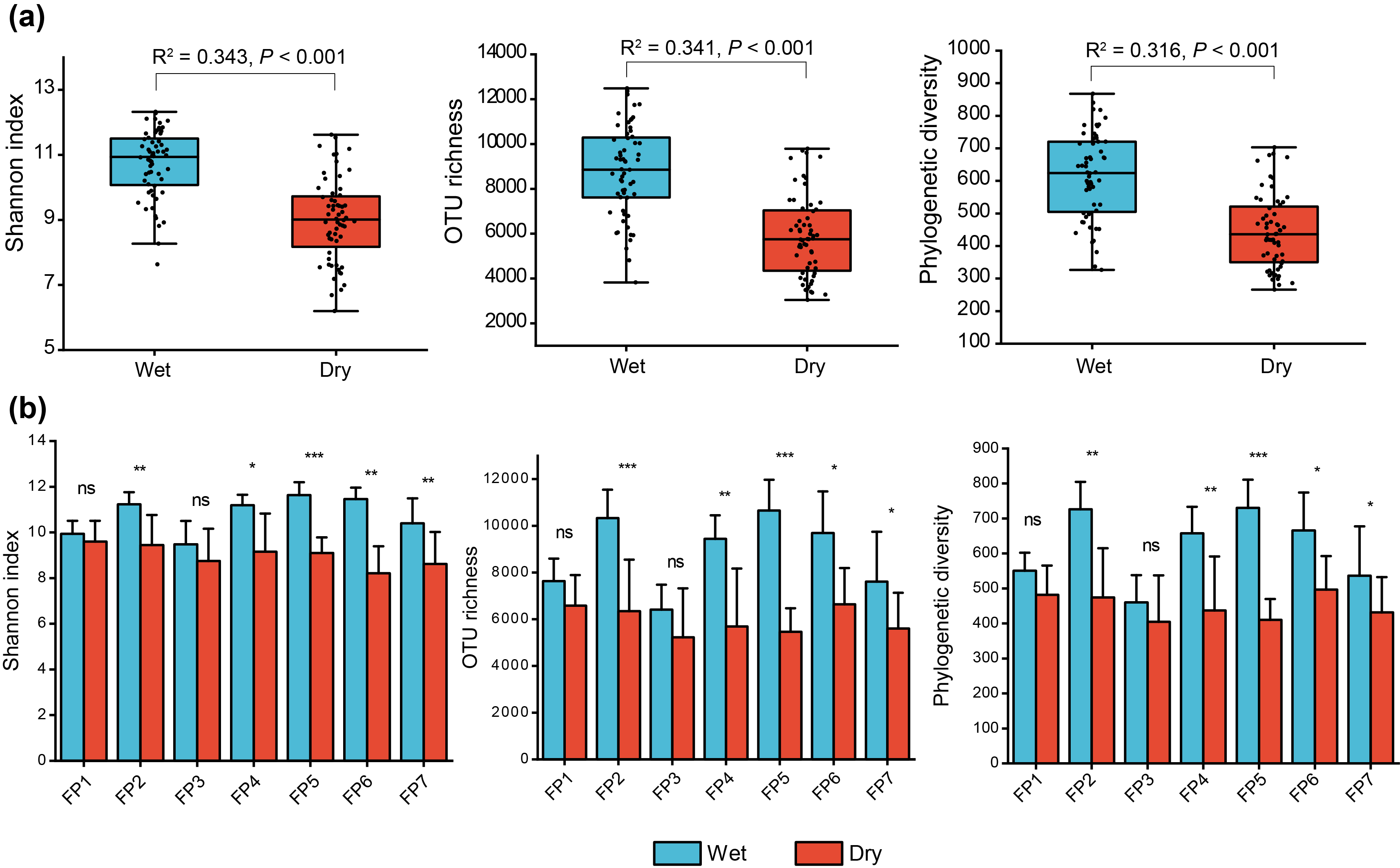

Supplement: FIG S2 [file msystems.01187-22-s0007.tif]

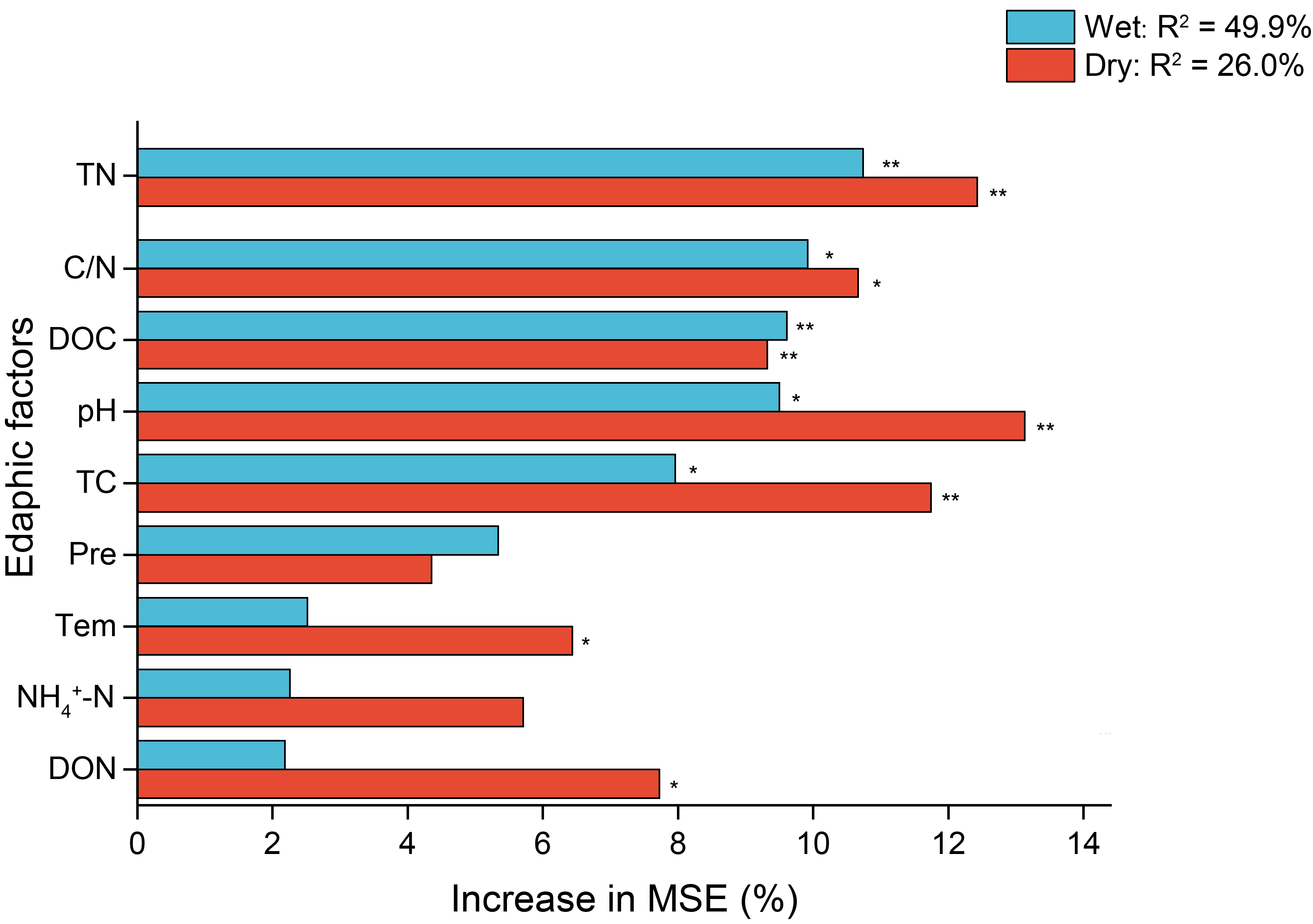

Supplement: FIG S3 [file msystems.01187-22-s0008.tif]

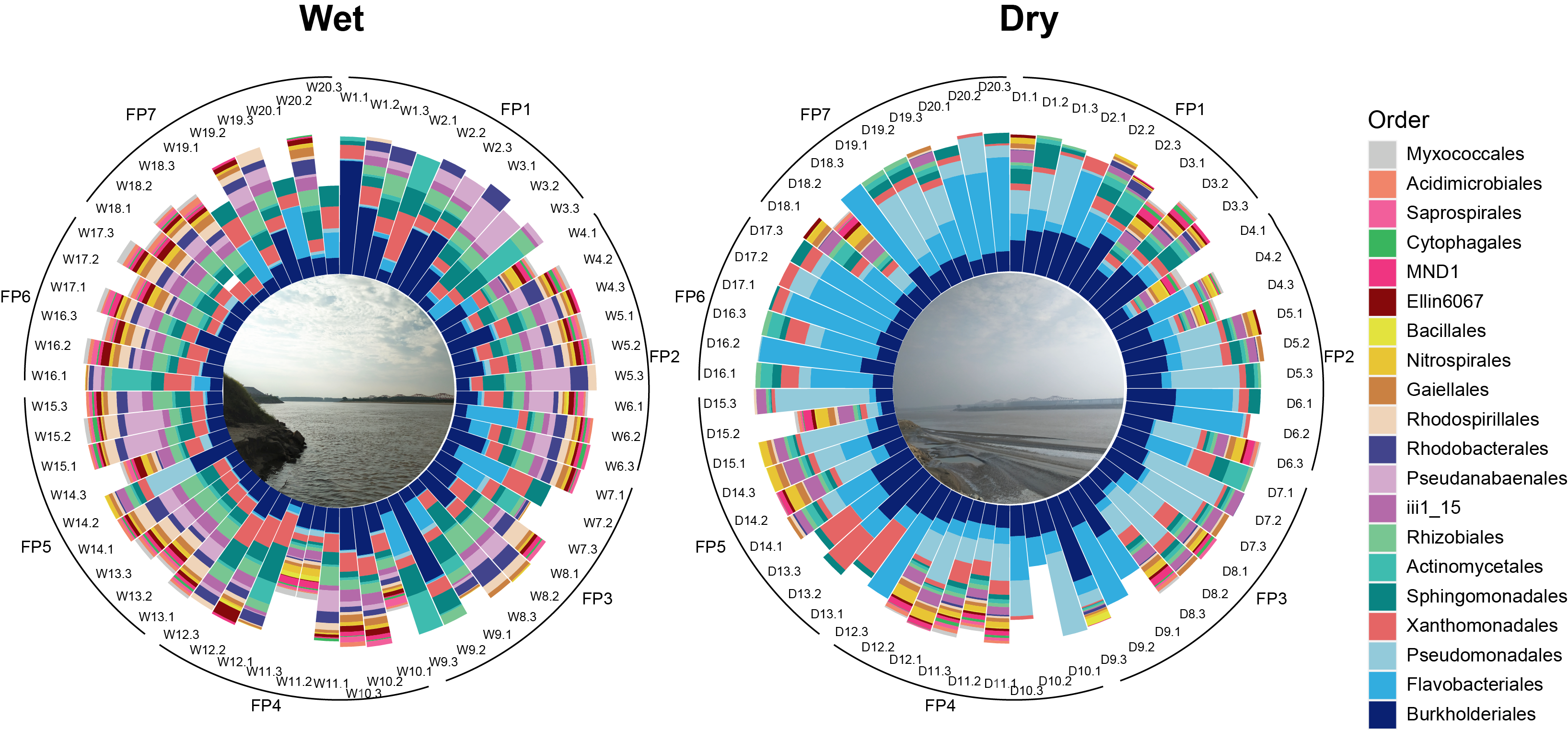

Supplement: FIG S4 [file msystems.01187-22-s0009.tif]

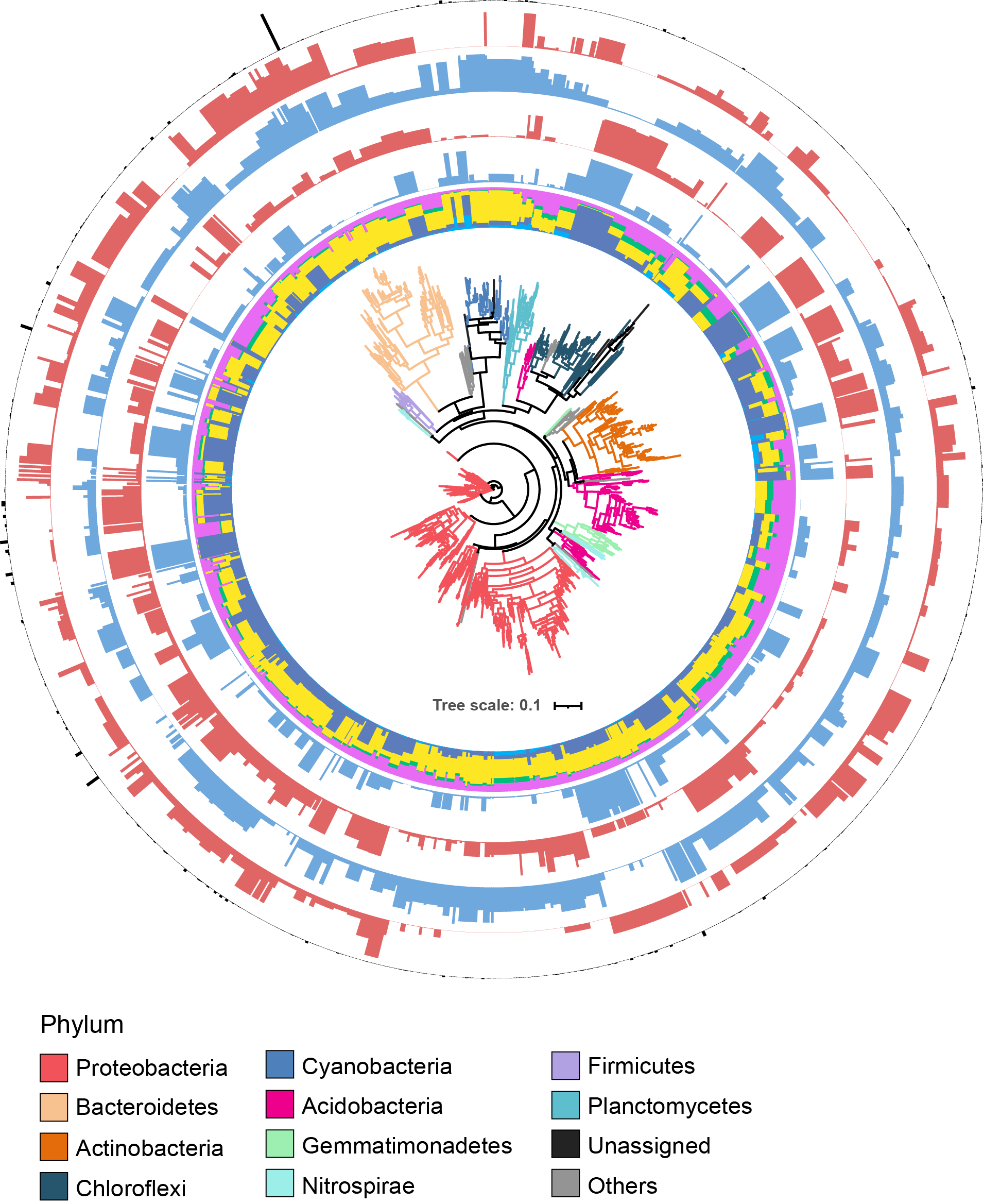

Supplement: FIG S5 [file msystems.01187-22-s0010.tif]

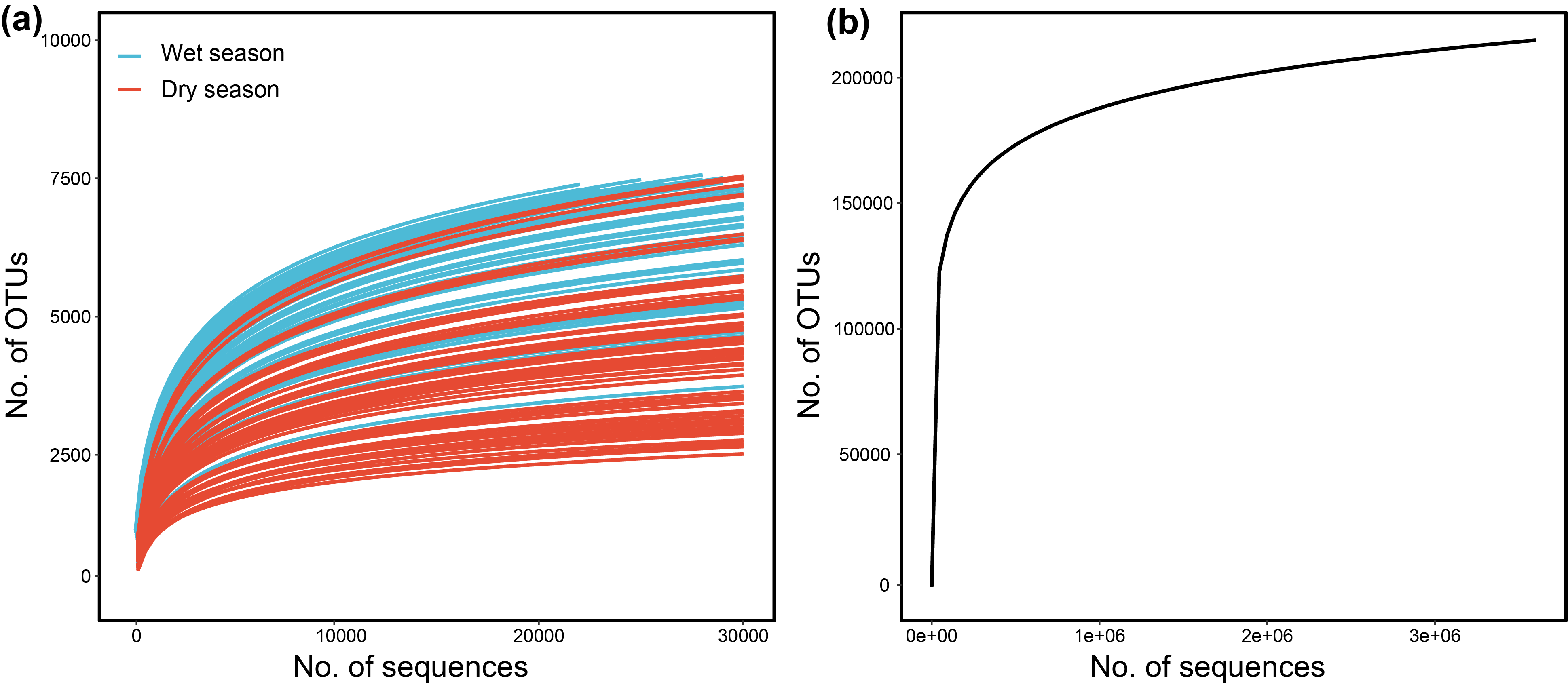

Supplement: FIG S1 [file msystems.01187-22-s0006.tif]
